# A cost-based equity weight for use in the economic evaluation of primary health care interventions: case study of the Australian Indigenous population

**DOI:** 10.1186/1475-9276-8-34

**Published:** 2009-10-07

**Authors:** Katherine S Ong, Margaret Kelaher, Ian Anderson, Rob Carter

**Affiliations:** 1 Centre for Health Policy, Programs and Economics, School of Population Health, The University of Melbourne, Carlton Victoria 3010, Australia; 2 Onemda VicHealth Koori Health Unit and Centre for Health and Society, School of Population Health, The University of Melbourne, Carlton Victoria 3010, Australia; 3 Deakin Health Economics, Public Health Research Evaluation and Policy Cluster, Deakin University, Burwood Highway, Burwood Victoria 3125, Australia

## Abstract

**Background:**

Efficiency and equity are both important policy objectives in resource allocation. The discipline of health economics has traditionally focused on maximising efficiency, however addressing inequities in health also requires consideration. Methods to incorporate equity within economic evaluation techniques range from qualitative judgements to quantitative outcomes-based equity weights. Yet, due to definitional uncertainties and other inherent limitations, no method has been universally adopted to date. This paper proposes an alternative cost-based equity weight for use in the economic evaluation of interventions delivered from primary health care services.

**Methods:**

Equity is defined in terms of 'access' to health services, with the vertical equity objective to achieve 'equitable access for unequal need'. Using the Australian Indigenous population as an illustrative case study, the magnitude of the equity weight is constructed using the ratio of the costs of providing specific interventions via Indigenous primary health care services compared with the costs of the same interventions delivered via mainstream services. Applying this weight to the costs of subsequent interventions deflates the costs of provision via Indigenous health services, and thus makes comparisons with mainstream more equitable when applied during economic evaluation.

**Results:**

Based on achieving 'equitable access', existing measures of health inequity are suitable for establishing 'need', however the magnitude of health inequity is not necessarily proportional to the magnitude of resources required to redress it. Rather, equitable access may be better measured using appropriate methods of health service delivery for the target group. 'Equity of access' also suggests a focus on the *processes *of providing equitable health care rather than on outcomes, and therefore supports application of equity weights to the cost side rather than the outcomes side of the economic equation.

**Conclusion:**

Cost-based weights have the potential to provide a pragmatic method of equity weight construction which is both understandable to policy makers and sensitive to the needs of target groups. It could improve the evidence base for resource allocation decisions, and be generalised to other disadvantaged groups who share similar concepts of equity. Development of this decision-making tool represents a potentially important avenue for further health economics research.

## Background

Global moves towards greater accountability of health care systems have increased the role of health economics and measures of efficiency in guiding resource allocation decisions. At the same time addressing the health needs of the whole population, particularly inequities in health, remains valued by most societies, and thus is an important objective for many health system decision-makers. For this reason, there is impetus for economic evaluation, the predominant tool of health economics, to expand its focus from maximising efficiency to also incorporate equity concepts [1-6]. In doing so, the results of such analyses would be made more relevant to the preferences of citizens, and criticisms regarding their practical applicability diminished. However, as yet there is no consensus about how this can best be achieved. Dissonance in opinion is largely driven by the normative nature of equity, and thus uncertainty in its precise specification, together with the difficulty in finding practical means for its measurement.

In this paper, the efficiency-equity debate from the health economics perspective is revisited. Definitions of efficiency and equity are briefly summarised, followed by an analysis of some of the currently available qualitative and quantitative methods to incorporate equity into economic evaluation. Specifying equity in terms of 'access' to health services, a cost-side equity weight for use in the economic evaluation of primary health care programs is then proposed, which we suggest overcomes some of the limitations of the other techniques. Based on the resources required to provide an equitable health service for the target group, the concept focuses on achieving equity in processes rather than outcomes, and is illustrated using the case study of primary health care delivery to the Australian Aboriginal and Torres Strait Islander (or Indigenous) population. In constructing the weight, we assert that the magnitude of health inequity is not necessarily quantitatively proportional to the magnitude of resources required to implement solutions which address these discrepancies. Other important advantages are that the method is relatively simple in construction, and is sensitive to the context and health beliefs of target groups. Therefore, this cost based equity weight provides a pragmatic and process-orientated, yet solutions relevant means of including equity alongside efficiency for disadvantaged sub-groups within priority setting exercises. It represents an alternative method for considering equity in the economic appraisal of health care services, to improve the evidence base upon which resource allocation decisions are made.

### Health economics and the efficiency objective

Health economics is primarily concerned with maximising efficiency. The aim is to maximise total benefit (often measured as health gain) with the available resources, and thus assist decision-making processes [7]. The need for regulation in achieving this objective results from the importance of social justice principles in the distribution of merit goods such as health care, together with the perceived failure of the competitive market to allocate resources efficiently within the health sector [8].

Economic evaluations can assist governments and other health care decision-making bodies make resource allocation decisions by indicating areas which have the potential to provide the most health gain, when resource constraints are present [7]. The cost-effectiveness ratios of alternative interventions are compared against each other, and decisions made according to criteria such as the available budget or predetermined thresholds.

However, economic evaluations tend to focus on maximising efficiency, with less regard to how these benefits are distributed amongst the population. Also known as distributional equity, this is an important policy consideration [4]. Maximising efficiency in isolation is not necessarily equitable, and in some cases may exacerbate disadvantage. As a result, the influence of economic approaches in many health care resourcing decisions is more limited than it might otherwise be [3].

### The need to incorporate equity and definitional variations

Health differentials are present and pervasive, both within and between different communities, societies, and geographical regions. Many of these differentials are large and affect those who are already disadvantaged [9]. Yet there is evidence that fairness and social justice are valued by most societies, to assist those who are in greater need [1,2]. Concern for equity was first notably articulated in the World Health Organization's Alma Ata Declaration of 1978 [10] which advocated 'health for all' using a comprehensive primary health care framework. The aim was to reduce health inequities using a preventive community-based focus to improve the health of the worst off. Although this framework has been critiqued as overly idealistic and has not been universally adopted [11], recent years have seen a resurgence with a refocus on universal health coverage and broad-based, context-specific primary health care [12]. Thus the pursuit of equity remains a significant guiding principle in public health.

Therefore, not only does efficiency or 'value for money' require consideration by decision-makers, but also the equity implications of resource allocations. For this reason, there is merit in incorporating equity concepts into economic evaluation techniques. Yet the complexity of the concept and difficulties in quantification mean that attempts at such endeavours are not simple.

One significant reason underlying continued debate about equity relates to definitional variations [13]. At the outset, distinction is made between inequality and inequity in health; it is generally agreed that health inequalities represent absolute differences in health status between individuals or populations, whereas health inequities represent inequalities which are considered unfair or unjust, particularly between different social groups [14-16]. In other words, health inequality is a positive or descriptive notion of what exists, while health equity is a normative notion of what should be. Beyond such broad definitions, however, there is no universal consensus as to which of the many measures of inequality is most relevant, and results differ depending on the measure used [16,17]. Moreover, there is no single definition of equity, as no explanation is appropriate to all contexts and populations.

The different interpretations of equity have been widely debated [18-27], yet ultimately all are normative propositions not amenable to scientific validation. As a result, there may never be definitive resolution of these issues. It has been suggested that the commonalities of these ideological approaches are greater than any theoretical differences [28]. In discussing different definitions of equity, Whitehead explains how what is 'unfair' may differ according to judgement, however the degree of *choice *afforded to those affected by health differentials is generally an important factor [9]. Moreover, it is the *opportunity *to achieve full health potential (via equality of access, utilisation or quality) that is required to achieve equity in health. Thus the focus should shift from debates about defining inequity towards achieving solutions [28]. It is not our intention to explore these arguments in detail, but what is required is that all assumptions be made explicit in each case to allow transparency of conclusions.

Therefore, for the purposes of this discussion, the following definitions have been selected:

#### Inequity in health

In using the term health 'inequity', we refer to health inequalities which are considered unfair or unjust. This involves poorer health of specific and identifiable population sub-groups, the fairness of which requires assessment on a case-by-case basis. It is in keeping with the definition of inequity specified by the International Society for Equity in Health, namely: 'systematic and potentially remediable differences in one or more aspects of health across populations or population groups defined socially, economically, demographically, or geographically' [29]. It is important that the distribution of inequity is specified, to allow comparisons to be made and measures taken to rectify these [14].

#### Equity in health care

With reference to health service delivery for these disadvantaged groups, the definition of equity we have selected to achieve fairness is to provide 'equity of access' to health services [22,27]. This is in contrast to other definitions which aspire to achieve equality of other aspects such as health, health care quality, or utilisation [9,18]. By specifying 'access', we imply equity of opportunity to obtain health care via the reduction or elimination of barriers, and this definition is in keeping with the social objectives of many societies to ensure appropriate health care is available to all [2].

Within health economics, equity can further be differentiated into horizontal and vertical equity concepts. *Horizontal equity *is defined as the 'equal treatment of equals', and this is the basis of most health economic evaluation processes where benefit (health gain) is deemed of equal value irrespective of to whom it accrues or their preferences for it [23]. Therefore, in this discussion, horizontal equity refers to 'equal access for equal need'. However, as horizontal equity does not take account of individual characteristics, it does not consider differences in pre-existing health status and thus differences in the 'need' for health care. Consequently where health disadvantage exists, *vertical equity*, defined as the 'unequal but equitable treatment of unequals', is important [23]. Using the access-based definition of equity selected for this discussion, vertical equity can therefore be specified as 'unequal but equitable access for unequal need' (hereafter referred to as 'equitable access for unequal need'). The implication is that preferential treatment is given to those deemed to be worse off, to enable improved access to health services. Although both horizontal and vertical equity concepts apply in this context, the focus of this paper is primarily on the goal of achieving vertical equity in the delivery of primary health care services. Therefore throughout the discussion that follows, subsequent references to equity relate to the vertical equity objective.

Achieving equity of access implies a *process oriented *definition of equity, as ensuring equitable 'access' to health services entails the removal of barriers that disadvantaged groups face in obtaining health care, including not only geographical barriers, but also cultural and financial impediments [22,27]. Therefore, there is a need for health service provision in a manner appropriate and non-threatening to the target population, using proximal and affordable comprehensive approaches.

As this point, it is worth noting that this discussion is focusing on incorporating equity into the economic evaluation of health interventions delivered from *health services*. As such, this is only one component of addressing overall inequities in *health *which have multiple and broad based social determinants both within and outside the health sector, and thus require correspondingly broad based solutions. For example, Wilkinson and Marmot describe factors such as the social gradient, stress, employment, and social supports, all of which impact on health [30]. However, the scope of this discussion is limited to the primary health care setting, in recognition that this is an important contributor to achieving overall health improvement.

In light of these selected definitional explanations, currently available methods to incorporate equity into economic evaluation and resource allocation techniques can now be analysed.

### Qualitative mechanisms for incorporating equity

Currently, economic approaches to health care resource allocation tend to incorporate vertical equity by way of qualitative judgements, either after economic analyses have been performed, or at the time of selecting options to be evaluated [1,31,32]. Thus equity is considered as part of the priority setting process in conjunction with efficiency, but is applied separately as a layer of judgement rather than being incorporated directly into cost-effectiveness ratios [33]. For example, the results of economic evaluations are frequently subjected to qualitative assessments of their performance according to certain dimensions, which in addition to equity, include the acceptability, feasibility and sustainability of interventions [32,34].

Qualitative equity judgements should be made explicit, to ensure the resulting decisions are transparent and accountable. Generally, this is achieved by way of 'pluralistic bargaining' and 'due process', whereby differences in opinion are resolved in an open and democratic manner [1]. This practice depends on the judgments of decision makers and other stakeholders, or the claims of the community and target groups [31,32]. Although the process can be made rigorous to a certain extent, these methods can be influenced by political ideology, the strength of arguments from special interest groups, and precedent [33,35]. The reality of vested interests is evidenced by the presence of advocacy groups and their lobbying tactics on policy makers, who in turn hold their own ideological beliefs which affect resource allocation decisions [35]. As a result, the outcomes of such processes may be ambiguous, and not as explicit as initially intended. Further limitations stem from disputes regarding who should be involved in the bargaining process, whose judgement should receive the most weight, and how the magnitude of any redistribution on equity grounds should be determined [31,36].

Difficulty in achieving consensus on what are often fluid issues can result in economic approaches focusing on allocation according to criteria other than equity, such as clinical effectiveness and efficiency, which are relatively concrete and amenable to 'objective' measurement [26,37]. Consequently, equity judgements may then be made by decision makers implicitly, with the reasoning behind them not subject to open scrutiny [3,36]. Therefore, as a result of its complexity, qualitative considerations of equity within economic evaluations are often given 'lip service' but not incorporated in a comprehensive and explicit manner.

### Quantitative methods of incorporating equity

As an alternative to qualitative methods, weighting mechanisms allow objectives such as vertical equity to be quantitatively incorporated alongside efficiency into cost-effectiveness ratios. The aim is to ensure economic evaluations encapsulate society's goal of assisting those with the poorest health. Equity and efficiency are combined in a quantitative manner, and in its standard form, numerical equity weights are applied to the *benefits or outcomes *accruing to those deemed to be in greater need. Benefit is commonly characterised as improvements in health status, using measures such as the quality-adjusted-life-year (QALY), which incorporates morbidity and mortality attributed to a condition [7]. The resulting equity weighted cost-effectiveness ratios therefore scale up benefits to the disadvantaged, producing more equitable allocations than those based on efficiency alone.

As a result, both efficiency *and *equity are considered as integral components of the primary quantitative analysis, and more consistent application of equity objectives is encouraged. Other advantages are that the opportunity costs of redistributions are made explicit [38], and guidance is provided on the optimal magnitude of allocation, as long as the relevant equity concepts are captured within the weight.

Equity weights are explored to a limited extent in the economic literature, and it is beyond the scope of this paper to provide a comprehensive review. In brief, the methods attempt to broaden the range of measured health outcomes to include social values of fairness by the application of equity weights to QALYs (often referred to as weighted QALYs) [3,4,6,38]. This may be done either by the direct weighting of QALYs according to predetermined criteria (for example, QALYs to certain target groups, such as the young, are worth more than QALYs to other groups) which are then maximised [4]. Alternatively, equity concerns may be incorporated within the health related social welfare function, which although more complex, pays more attention to ensuring equity in the distribution of outcomes [4].

The construction of equity weights is dependent on the definition of equity selected. Some weights, such as the age weights developed by the World Bank, endeavour to reflect the social value of an individual's contribution to the rest of society [38]. However, most of the economic discourse on weights reflects society's concern for equity in how resources are distributed amongst individuals, and a desire to give preference to those who are worse off [3]. Further discussion then focuses on the issue of which of the many components of equity should be incorporated into the weight, and what magnitude each should contribute [3,4,6,18,38-40]. For example, Williams' 'fair innings' argument weights health gains in favour of the young [38], while Nord's 'cost-value analysis' (CVA) advocates weights that capture societal concern for the severity of illness and reluctance to discriminate against those with limited potentials for health gain [6]. Meanwhile, Stolk's 'proportional shortfall' method combines the fair innings and severity of illness arguments [3]. All methods aim to address equity via the weighting of QALYs.

However, although relevant to definitions of equity selected for their own purposes, these weighting approaches have some limitations in their construction with regard to the definition selected for this discussion based on achieving equitable access for those with greater needs.

First, these methods focus on distributive justice and apply weights to the *outcome *or *benefit *side of the economic equation, when in fact attention to processes may be more relevant [3,6,38]. In attempting to achieve 'equity of access', a focus on processes means that equity is considered in the *ways in which health services are delivered *rather than on the end results, and there is greater consideration of improved access to services. Using this definition, application of equity weights to health *outcomes *is less relevant as there is no direct consideration of 'access'. Although increased levels of benefits may indirectly imply that access has been improved, there is no *direct *assessment of the processes by which these outcomes are achieved.

As noted by Mooney, Wiseman and Jan, measurement of outcomes also becomes problematic when different constructs of health are held by different groups [23,26]. In such cases, distributive justice based on outcomes is difficult to achieve as outcomes are not valued equally by all. For example, QALYs arguably take a narrower perspective of health defined in terms of individual morbidity and mortality, and consequently may not fully capture broader concepts such as spiritual, community and environmental impacts, which are valued equally by some populations [41]. Therefore, a fair distribution of QALYs based on societal values may not necessarily be fair to all groups if they do not value QALYs similarly [6,38]. In such cases procedural justice or fairness in processes (not only of decision making, but in the processes of achieving the stated goal or outcome) becomes more relevant. Economic evaluation by definition needs to use some form of outcome measurement, as the definition of cost-effectiveness is cost per unit of *effect*. So although there is a requirement for the use of outcomes, there is also scope to increase the use of processes in determining a more equitable result. As described by Mooney and Jan, the process of applying cardinal equity weights can allow extension of the concept of equity to include the valuation of processes as well as health outcomes [23].

The second limitation is that the size of outcomes-based weights is based on theoretical judgements regarding the dimensions and degree of inequity. For example, the World Bank's age weights are generated from a derived exponential equation [42]; Williams' 'fair innings' weights are based on mathematical formulations of the social welfare function [38]; while Nord's CVA relies on multiple judgements based on the person-trade-off technique [6]. Given that there are many different dimensions of equity, all valid and valued by society to varying degrees, there is difficulty in specifying such concepts accurately within a single weighting measure [4]. Moreover, the assumption that the magnitude of inequity, should this be measured, is also an indication of the level of resources needed to address this is debatable [37]. Finally, at a pragmatic level, we contend that such theoretical derivations may be cognitively challenging, particularly for non-economist decision-makers who are often charged with understanding and acting on the results.

Weighting techniques are still very much experimental, with no specific form universally accepted to date [4]. Therefore, scope exists for further exploration of these concepts and methods, including attempts to develop alternative methodologies which are sensitive to the needs of target populations while addressing some of the limitations outlined. Therefore, in the reminder of this discussion, we explain the conceptual basis of a cost- or process-based equity weight for use in the economic evaluation of primary health care services. This is illustrated using the case study of the Australian Indigenous population.

## Methods

### Cost-side equity weights - a proposed alternative

**Figure 1:**
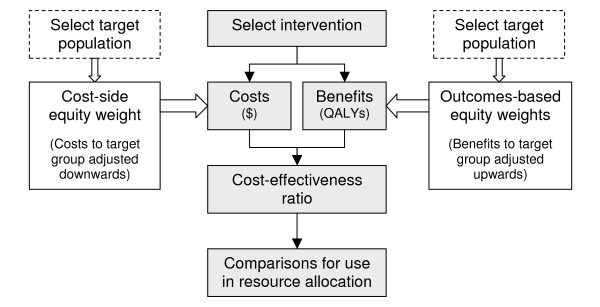
**Simplified economic evaluation schema with the application of equity weights**. The basic economic evaluation framework (shaded) involves selection of interventions or programs to evaluate, followed by determination of their incremental costs (in monetary units) and outcomes or benefits (often using health state measures such as the QALY) compared to current practice. From this data, the incremental cost-effectiveness ratio (ICER) can be calculated as the net cost per unit of benefit, and the results then compared with the ICERs of other interventions for use in resource allocation and decision-making. Equity weights can be applied to the analyses of selected target populations who are deemed to be worse off. Conventional outcomes-based equity weights apply a weight to the benefits of the intervention, by weighting up the QALYs attributed to disadvantaged groups. The proposed alternative is to apply a cost-side weight to the costs of the intervention, based on equitable *processes *of health service delivery.

Table 1 provides a summary of the methods to incorporate vertical equity into economic evaluations, including their benefits and limitations. In contrast to the described 'outcomes-based' weights, we propose a cost-side equity weight for use in the economic evaluation of primary health care services. This weight is based on the 'processes' of health service delivery in keeping with the selected definition of equity 'equitable access for unequal need'. Differences between the two weighting approaches can be illustrated by way of the basic economic evaluation framework (Figure 1).

**Table 1:** Summary of methods to incorporate vertical equity into economic evaluations

	**Qualitative methods**	**Quantitative methods**	
		**Outcomes-based equity weights**	**Cost-based equity weights**

**Basis for equity adjustment**	Decision-maker and/or stakeholder assessment of impact on equity	Weighted QALYs - by direct weighting or characterisation of the social welfare function	Costs weighted based on additional resources to provide improved access to health services

**When performed**	Before or after calculation of cost-effectiveness ratios	Incorporation into the benefits side of cost-effectiveness ratios	Incorporation into the cost side of cost-effectiveness ratios

**Examples**	Pluralistic bargaining ACE 2^nd ^stage filters [34]	Fair innings [38] Cost-value analysis [6] Proportional shortfall [3]	Cost side equity weight described in this paper

**Main advantages of approach**	Less resource intensive than quantitative methods Quick and doable with existing personnel	Explicit equity assumptions and judgements Guidance on magnitude of resource redistributions based on social welfare	Explicit equity assumptions and judgements Guidance on magnitude of resource redistributions based on solutions to inequity Equity considered in health care processes rather than outcomes Specific to context and definition of health for target group Basis of weight simple to conceptualise Comparable across different target groups

**Main limitations of approach**	Equity judgements may be implicit No guidance on magnitude of redistributions	Generally do not consider equity in processes of health care delivery Not sensitive to differing preferences of target groups Assumption of proportionality between magnitude of inequity and its solutions Complex theoretical basis	Weight based on 'improved' rather than equitable access May be resource intensive to construct Dependent on a 'best practice' health service model being available for the target group May lead to perverse incentives (i.e. reward inefficiency) Untested in real policy decision contexts

There are two key steps to developing an equity weight in this context. First those populations deemed to be worse off and with greater health needs have to be identified as target groups (for whom distribution is considered inequitable), based on the magnitude of health inequity between different groups. These are the groups to whom the weight will be applied. Second, the magnitude of resources to be reallocated to these groups to achieve more equitable access has to be determined, to establish the magnitude of the weight [37].

Identification of target groups is required in all forms of equity weighting, by elucidating and measuring the parameters where inequalities are present and considered inequitable. This has been relatively well covered and explored in the literature, where numerous methods to assess levels of health inequalities have been developed, such as the Gini coefficient and the concentration index [17,43-45]. Whether health inequalities are deemed inequitable may be determined by inbuilt components of these measures, by the external application of judgements based on empirical studies and formulations of the social welfare function, or by other techniques such as community preferences and claims [46]. These measures will not be discussed here. The magnitude of the proposed cost weights is not dependent on the method used to select and measure inequity amongst target groups, but rather on how the inequity should subsequently be addressed.

Once target groups with greater needs have been *identified *using one of these established measures of inequity, we propose an alternative method of specifying the *magnitude *of the equity weight based on the resources required for redress. This area has been recognised as one where the evidence base remains relatively thin and further research is required [44,47].

It is proposed that the size of the equity weight be calculated by measuring the costs of providing specific types of health interventions to the target population via appropriate and 'best practice' mechanisms which are preferred by the community. The method of health service delivery would be congruent with the definition of health relevant for each target group, and should be selected using the best available evidence, along with communitarian claims or entitlements, or community preferences [23], as to what constitutes a fair and equitable method of health service delivery for that group. Provision of health services in this manner will assist overcoming barriers to accessing primary health care, and can therefore be expected to improve equity. These costs can then be compared to the costs of the same interventions delivered from a 'baseline' mainstream health care service. The type of baseline primary health care service selected (whether general practitioner or community health service based) would be less important than stating a clear definition, to allow its consistent application. The cost weight would subsequently only be applied in the context of this same baseline comparator.

This comparison between intervention delivery via targeted and baseline health services will allow differences in the *processes *of health service delivery between the two alternatives to be quantified, in order to achieve equity via procedural justice. The magnitude of the weight would depend on the ratio between the two, and, assuming greater costs of targeted services, would thus be a measure of the additional resources required to address equity in primary health care when compared to the base case. This weight could then be applied to the costs of similar types of interventions directed at the same target population as an equity adjustment. The process will be repeated for different intervention types, thus producing a set of 'reference weights'. As long as the same baseline service is used as a comparator, the magnitudes of such weights would be comparable, both within and across different target groups, and thus able to be used in resource allocation decision-making.

Once developed, the reference weights can be applied alongside efficiency objectives within economic approaches to health care resource allocation. In practice, the weight would not necessarily result in an equal distribution of 'health outcomes', but would ensure greater equity of access by not adversely penalising interventions which cater for the specific needs of those who are worst off, albeit at greater cost. The results could thus allow policy and decision-makers to allocate resources according to economic evidence which not only considers 'value for money', but also equity for disadvantaged groups. Although we have used the example of primary health care, a similar process could also be used for secondary and tertiary health care services.

### Development of cost-based weights for the economic evaluation of primary health care services - illustrative case study of the Australian Indigenous population

The development of this cost-side equity weight is illustrated with reference to the Australian Indigenous population as a case study. This is a group for whom health inequities clearly exist irrespective of which measure is used [47]. For the period 1996-2001, life expectancy for this group was more than 17 years less than their non-Indigenous Australian counterparts, standardised mortality ratios were three times the rate, and infant mortality levels fifty percent higher [48-50] (Table 2). Trends indicate that health differentials in some areas are increasing rather than decreasing between the two groups [51], and reasons for continued health disadvantage include systemic environmental and socio-cultural factors, along with historical, cultural and institutional barriers to accessing adequate health care [47,52-58]. In particular, diminished primary health care access is illustrated by lower uptake of preventive interventions such as immunisation and screening programs in the Indigenous population [59-62], resulting in increased presentations at tertiary centres at more advanced stages of potentially avoidable disease [63]. Thus, the health status of the Indigenous population continues to be inferior to that of the general Australian population in a way that can be considered inequitable [64].

**Table 2:** Selected indices of Indigenous and general Australian population health status and access to health services

**Health index**	**Indigenous** **Australian population**	**General** **Australian population**
Life expectancy - Females (1996-2001) [50]	65 years	82 years

Life expectancy - Males (1996-2001) [50]	59 years	77 years

Perinatal death rate (2003-2005) [50]	15.7 per 1000 births	10.3 per 1000 births

Standardised Mortality Ratio - Males (2001-2005) [50]	3.0	1.0

Standardised Mortality Ratio - Females (2001-2005) [50]	2.9	1.0

Immunisation rate at 12 months of age (2000) [60]	72-76%	90-94%

Cervical cancer screening rate in Northern Territory (1997-98) [61]	34%	64%

Therefore, it is clear that the Indigenous population is a target group deserving immediate priority on vertical equity grounds [26], and this concern has been exemplified by recent Australian Government policy imperatives [65,66]. Additional resources are required to redress this situation, however, to date improvements have been marginal, and several reports have concluded that much more is required [47,67,68]. Yet recommendations are commonly presented as general funding targets, and specific guidance is required as to where investments can best be positioned to effectively tackle inequities at reasonable cost.

The health needs of the Indigenous population need to be understood in terms of the Indigenous definition of health, which in the National Aboriginal Health Strategy (NAHS) of 1989 was defined as:

Not just the physical well being of the individual but the social, emotional, and cultural well-being of the whole community. This is a whole-of-life view and it also includes the cyclical concept of life-death-life [69].

Indigenous concepts of health not only encompass the absence of individual illness, but also embrace the wider community context. This includes whether treatment delivery processes are congruent within this broader setting. In addition, the definition of equity based on equitable access selected for this discussion is relevant to this group, as it is the same definition adopted by the National Aboriginal Health Strategy, a policy document developed by and for the Australian Indigenous population [70].

Having identified that the Australian Indigenous population is a group in greater need and establishing the context of health disadvantage and perceptions of health experienced by this group, the next step is to quantify 'equitable access' to determine the magnitude of the weight. With respect to primary health care, qualitative research points to provision via community based and controlled health services (Aboriginal Community Controlled Health Services, or ACCHSs) as a significant means by which many of the access barriers faced by Indigenous people can be overcome [69]. ACCHSs are strongly grounded in the comprehensive primary health care philosophy, which comprises in addition to clinical care, a strong emphasis on illness prevention, health promotion and community support within a culturally safe environment [58,71-73]. This service type would therefore seem to engender the appropriate processes upon which equity of access could be quantified. We are currently in the process of devising a 'template' of how primary health care delivery via ACCHSs differs from mainstream services in Australia for use in economic evaluations (for a brief description of this work see Additional file 1: The Indigenous Health Service Delivery (IHSD) Template).

At this point it is worth briefly noting that in performing economic evaluations, there is a need to consider both the costs and the outcomes (or benefits) of an intervention. In considering outcomes, it is important that differences in the underlying baseline disease risk of target populations and effect sizes due to interventions delivered from different health service types are taken into account. This is incorporated into the economic evaluation process via economic and epidemiological modelling, which focuses on determining the effects of interventions by extrapolating evidence on changes due to the intervention (for example, taken from clinical trials) to calculate changes in final outcome measures (such as QALYs). However, as this paper focuses on the development of a cost-based weight for *subsequent use *in economic evaluations, the focus is on cost differentials. Differences in effect size as the result of an intervention are part of a separate modelling process and are not relevant to the construction of the weight. Therefore, although important in the overall economic evaluation process, differences in effect size are not considered in the remainder of this discussion.

A numerical example of how cost-weights for the economic evaluation of primary health care services could be developed for the Indigenous population, based on the processes of care, and then employed is provided as an additional file (see Additional file 2: Case study - development and application of a cost-side equity weight for the Australian Indigenous population).

## Results and Discussion

The contribution of health economics within health care resource allocation decision-making is growing [3]. In part, this growth can be attributed to globally increasing health care expenditures caused by the ageing of populations; the development of new technologies; and changing expectations of what the health system can deliver, set against a backdrop of limited available funds [1]. In addition, the growing burden of chronic non-communicable diseases has led to increased ongoing health care costs and a call to reorient health care resources towards prevention, partially pushed by economic imperatives [74]. The expanding role of economics is illustrated by the Pharmaceutical Benefits Scheme (PBS) in Australia, whereby pharmaceuticals must show evidence of cost-effectiveness prior to being eligible for public subsidy [75]. With this increasing usage, arguably comes a responsibility for economic methods to pay greater heed to social values such as equity concerns for those who have poorer health. Both efficiency and equity objectives are important in ensuring resource allocation formulae remain relevant and appropriately measure societal welfare.

We have proposed a cost-based weighting mechanism to incorporate vertical equity alongside efficiency in the economic evaluation of interventions delivered from primary health care services, based on ensuring equity of access. This method uses established measures of health inequity to identify target groups in greater health need, and then uses measures of the processes of health service delivery to determine the magnitude of resources required to provide equitable access to primary health care services as a cost-weight. We assert the social value of this weight lies in the normative concept that cost-effectiveness ratio distortions are corrected to reflect society's objectives of addressing inequity in a solutions-based manner. While still based firmly on social concern for equity in determination of target groups and best practice models of health care, there is less dependence on use of social values to determine the magnitude of the weight itself.

The application of this cost weight is in addressing vertical equity in the economic evaluation of interventions targeting disadvantaged population sub-groups. The aim is to assist decision-makers allocate resources in a manner such that both efficiency and equity objectives are fulfilled.

### Non-proportionality of 'need' and 'access'

Two points should be noted regarding the derivation of this cost based equity weight. First, we take the view that although commonly used in funding formulae (such as the Resource Allocation Formulae, RAF [31]), the magnitude of measured health *inequality *is not necessarily the same as the magnitude of resource redistribution required to address the discrepancy [27,47,76]. Moreover, the level of determined *inequity *(or need as measured by the social welfare function), which is used by some other weighting methods, is again not the same as the magnitude of resources required for redress. This arises because there is no reason that solutions to inequity (in this case, achieving equitable 'access') are necessarily proportional to the magnitude of inequity itself (measured as 'need'). Mooney questions the existence of a cardinal relationship between, for example, Standardised Mortality Ratios (SMRs) and the allocation of resources [37], and the same argument could be made for other measures of inequity. Simple and cheap solutions may be required to address large inequities in health, or conversely small inequities may require a large reallocation of resources. However, the magnitude of health inequity is still an important factor in determining target groups who are disadvantaged, and also for prioritising the need for action to redress these.

The second issue is that in focusing on improving equity in health care, this only deals with one component of overall inequities in health. Full solutions require much broader inter-sectoral action which tackles the underlying social determinants of health [9,10]. Thus it cannot be expected that addressing inequities in health care will solve all health inequities, and a proportional relationship between the two cannot necessarily be assumed.

In taking this position, the conceptual link between health status inequities and health care solutions is not being denied. Rather, it is disputed whether this relationships is necessarily a quantitative one-to-one. The 'value' incorporated in this approach lies in equitable redistribution to address barriers target groups face in obtaining health care, with solutions pragmatically based on the processes of equitable health service delivery rather than theoretical formulations of social welfare based on preferences. Although this is not how value is measured in the orthodox economic sense, it is a measure of valued processes of health care by the target group.

In separating the measurement of inequity from the magnitude of resources required for redress, the notion of vertical equity as 'equitable access for unequal need' is deconstructed. Measurement of 'unequal need' is based on health differentials or *outcomes*, while determining the magnitude of the weight by providing 'equitable access' relies on specifying the *processes *of health service delivery. In rationalising these discrepancies, it could be argued that the two steps represent different purposes: first, the identification of inequity and therefore target groups, and second, proposed methods to rectify this. Because different purposes are represented, the use of different concepts is justifiable and congruent with overall objectives.

### Benefits of cost weighting

While maintaining the benefits of other quantitative methods, one advantage of this cost weight lies in its construction as a process- rather than outcomes-based approach, more in keeping with the selected definition of equity based on achieving equitable access to health services. Moreover, specification of the way health care services are delivered provides a practical mechanism to determine equitable access or the *magnitude *of the weight. By basing the magnitude of the weight on the resources required to provide an appropriate and equitable health service, not only is the method of determining the size of the weight made transparent, but there is also less ambiguity for decision-makers, as processes of service delivery may be relatively easy to conceptualise compared to mathematically derived formulae or other qualitative judgements regarding the social welfare function.

Primary health care delivery to the Australian Indigenous population has been used as a case study to illustrate the development of the weight, however the technique would be generalisable to other disadvantaged groups who share a similar definition of equity. Furthermore, the use of processes of health service delivery allows for different definitions of health which may be held by distinct target groups to be incorporated into the weight making it context specific, and this averts the need to use a broader definition of health which is more general and less relevant to those to whom the equity weights seek to address. As long as the same baseline health service is used as a comparator to determine its magnitude, such weights could then be used to ensure uniform assessment across disparate programs and target groups in resource allocation decision-making.

### Limitations

The proposed weighting mechanism is not without its weaknesses, however. There are limitations in attempting to devise weights which apply to whole population subgroups, which are often heterogeneous across communities and locations. In such cases, separate weights could be derived for sub-segments of the group, however, increasing the specificity of the measure would need to be balanced against the amount of resources required for its development and the practicality of performing multiple case studies. Should broader more general weightings be used, then results would be indicative of resource requirements in particular health services, but would not be prescriptive.

Similarly, development of the cost weight requires that there is an available model of 'best practice' primary health service delivery (such as ACCHSs for the Australian Indigenous population) upon which to base the weight. There may not be such a model readily available for all target groups. It could be argued that the absence of such a model would represent a major barrier to reducing health inequities for this group, and would therefore warrant further research as a priority. In the meantime, many of the principles incorporated into ACCHSs such as community direction, multidisciplinary care and a focus on health promotion and disease prevention make up an important component of the philosophy of primary health services targeting disadvantaged groups [77-81], and a generic model such as community health services could be tailored to each specific case.

Although resource use is relatively easy to measure and provides a concrete justification for the size of the weight, it is acknowledged that there will still be a role for judgement in such measures, for example as to what exactly constitutes an 'appropriate' health service for the target population. Therefore, the method described is still to a certain extent subjective, however judgements made will be more explicit, have greater input from target groups, and be based upon practical real-life examples.

It is also recognised that it may be difficult to prove that a certain method of health service delivery (such as ACCHSs in this case study) provides *equitable access *for target groups, and rather, it is *improved access *that is in fact being measured. It therefore follows that the equity weight will not, in effect, weight health care resources in a completely equitable manner, but in a more equitable manner than is current practice, and in the best way possible given the current evidence. This issue is not unique to this cost weight however, and as described by Culyer: '...The fact that there will never be sufficient information for the judgements [about equity] to be reached with absolute confidence should not stand in the way of policies which push the system in the direction that equity demands. The perfect should not be allowed to become the enemy of the merely good.' [13].

While the weights encourage equitable assessment of interventions *delivered *in an appropriate manner, whether the interventions *themselves *are equitable remains an issue. For example, interventions which are broad based and community directed are generally preferred by Indigenous populations over those which are vertical or 'top down', however this preference will not be captured by the weight. Therefore, there will still be a role for qualitative judgement regarding which interventions should be selected for evaluation in the first place, and these judgements are best directed by the target groups.

Another limitation is that basing the magnitude of the weight on preferred methods of health service delivery could lead to perverse incentives for inefficiency, as greater costs of health services will lead to greater cost-weights being allocated (i.e. make the results more favourable for inefficient services). It will be important that this is prevented, by only constructing weights using 'best practice' models of health care grounded in robust evidence.

In addition, use of the ratio of different health service costs to determine the magnitude of the weight assumes that the costs of targeted services are greater than those of the baseline service. This is an important assumption as, if the costs are in fact lower, the resulting weight would direct resources *away *from the target group. In reality, this scenario is unlikely to occur, as services to disadvantaged groups generally require more resources, not less. However, it remains a theoretical possibility and thus construction of the weight should only proceed once greater costs requirements for the target group have been established. Once again, this limitation is not unique to this case however, and also applies to other outcomes based weighting mechanisms.

### Methodological issues

It is acknowledged that the process of constructing cost based equity weights will require a large additional amount of data to be collected in the economic evaluation process. In effect, an additional economic evaluation will need to be performed for each intervention affecting each target group separately. However, once the initial data has been collected for the differences in costs and effects of treatment for each group, this can be applied more simply to the evaluation of subsequent interventions. It is argued that such additional effort is feasible, and is necessary to address equity in a meaningful way, irrespective of what method is used to incorporate it (whether qualitatively or using other weighting mechanisms).

As part of this analysis, two important caveats should be noted. First, economic appraisal is different from financial appraisal, particularly when using equity weights. Thus weighted intervention costs used in economic analyses will require conversion back to their unweighted form when determining the size of funding allocations to avoid underestimation. Second, it is important to note that formula-based decision-making is not the intended purpose of this weight. Qualitative judgements, by policy makers, target groups and wider society, will always have a significant role in resource allocation to account for the unique circumstances of each situation. However, equity weights in conjunction with efficiency results from economic evaluations could provide valuable input and greatly improve the evidence base upon which such decisions are based.

Finally, greater use of economic approaches in the allocation of resources for Indigenous health remains contingent on further development of methods to successfully incorporate equity. This discussion has been led by Mooney, Jan, Wiseman and colleagues, who have advocated distribution based on 'claims' for resources according to certain dimensions [26,27,46,82,83]. Such claims are determined by the community, and extension of these principles to funding formulae is explored in work by Henry and Houston [76,84,85]. These methods share a similar underlying philosophy of the need to focus on processes rather than outcomes in order to achieve equity relevant to the population involved. The method described here adds to the discussion by suggesting use of health service delivery processes, as a practical alternative to quantify the value or magnitude of weighting redistributions applied to the cost side of the economic equation.

## Conclusion

The cost-based weighting mechanism proposed in this paper provides a process- rather than outcomes-based method of quantitatively incorporating vertical equity objectives alongside efficiency objectives in economic approaches to health care resource allocation. As a quantifiable measure, it encourages equity concerns to be considered in a consistent, explicit and transparent manner, while the use of health service delivery features encapsulates the process-based definition of equity based on achieving 'equity of access' to health services which is a common policy approach. We would argue that an important contribution of this technique is a practical means to determine the magnitude of the equity weight based on solutions to health inequity rather than the measured inequity itself, while remaining grounded in theory. The method aspires to be relevant to community preferences and comprehensible to policy-makers. Despite ensuring that analyses are context specific, reference to the same baseline service in construction of the weights allows standardised comparison of interventions targeting different groups, and therefore they may be of value in allocative efficiency decisions.

To date, we are unaware that any such cost derived weighting mechanism exists for use in economic approaches to resource allocation. In our view this research direction is worth investigating to broaden the methods by which vertical equity concerns are incorporated in contexts where an access-based definition of equity is relevant. A prototype set of weights is currently being developed for use in evaluating preventive interventions targeting Indigenous Australians; a group for whom health inequities clearly exist, have persisted over time, and where there is an urgent need for alternative approaches to determining priorities. The implications for policy are the development of an aid to decision-making that enables both efficiency and vertical equity concerns to be effectively captured within cost-effectiveness ratios.

## Competing interests

The authors declare that they have no competing interests.

## Authors' contributions

KO performed the literature review, conceptualised and developed the theoretical ideas, and wrote the manuscript. MK contributed to the theoretical ideas and helped to draft the manuscript. IA contributed to the theoretical ideas and helped to draft the manuscript. RC oversaw the project, contributed extensively to developing the theoretical ideas, and helped to draft the manuscript. All authors read and approved the final manuscript.

## Supplementary Material

Additional file 1 **The Indigenous Health Service Delivery (IHSD) Template**. A brief outline of work in progress towards development of a template detailing differences in primary health care service delivery between Aboriginal Community Controlled Health Services (ACCHSs) and mainstream general practitioner services in Australia, for use in economic evaluations. Click here for file

Additional file 2 **Case study - development and application of a cost-side equity weight for the Australian Indigenous population**. A numerical example of how cost-based equity weights for the economic evaluation of primary health care services could be developed for the Indigenous population, based on the processes of care, and then employed. Click here for file
