# Supplementary material for: A cost-based equity weight for use in the economic evaluation of primary health care interventions: case study of the Australian Indigenous population
Source: Int J Equity Health. 2009 Oct 7;8:34. doi: 10.1186/1475-9276-8-34 (PMC2768712; doi:10.1186/1475-9276-8-34)
Supplement: Additional file 1 — The Indigenous Health Service Delivery (IHSD) Template. A brief outline of work in progress towards development of a template detailing differences in primary health care service delivery between Aboriginal Community Controlled Health Services (ACCHSs) and mainstream general practitioner services in Australia, for use in economic evaluations. [file 1475-9276-8-34-S1.PDF]

The Indigenous Health Service Delivery (IHSD) Template

The objective is to develop a template of ‘best practice’ primary health care service delivery for the Australian Indigenous population based on Aboriginal Community Controlled Health Services (ACCHSs), for use in economic evaluations. Components and activities that differentiate ACCHSs from mainstream general practitioner (GP) based health services have been identified, measured and valued.

Components identified include:

- Differences in the structure of consultations with a multidisciplinary focus, including the employment of Aboriginal Health Workers
- Greater recruitment, training and reporting requirements
- An increased focus on population health activities with collaboration outside the health sector
- Governance via a community management board
- Widespread provision of transport services
- Provision of services to a large remote population including outreach and emergency services

Measurement and valuation of these components has been performed (not detailed here), and differences collated as the additional costs of providing a patient consultation at an ACCHS compared to a mainstream GP practice. Provisional results indicate an average cost of $113.33 (AUD) for a basic consultation at an ACCHS compared $30.85 for a mainstream GP consult (2003 values). Uncertainty in these values has been determined but is not shown.
