# Supplementary material for: A cost-based equity weight for use in the economic evaluation of primary health care interventions: case study of the Australian Indigenous population
Source: Int J Equity Health. 2009 Oct 7;8:34. doi: 10.1186/1475-9276-8-34 (PMC2768712; doi:10.1186/1475-9276-8-34)
Supplement: Additional file 2 — Case study - development and application of a cost-side equity weight for the Australian Indigenous population. A numerical example of how cost-based equity weights for the economic evaluation of primary health care services could be developed for the Indigenous population, based on the processes of care, and then employed. [file 1475-9276-8-34-S2.PDF]

Case study – development and application of a cost-side equity weight for the Australian Indigenous population

Selection of target group:
The Australian Indigenous population is identified as being disadvantaged, and therefore in greater need, using established measures of health inequity. The magnitude of inequity helps to prioritise the need to redress this. An equitable primary health care service, based on achieving equitable access, is delivered via the Aboriginal Community Controlled Health Services (ACCHS) model.

The intervention:
For a hypothetical pharmacological treatment, the ‘baseline’ service involves consultation with a medical practitioner who prescribes and monitors treatment within the mainstream health care system. If the cost of the medication is taken to be $200 per annum, with two GP consultations at $30.85 each (see Additional file 1), in this simplified example, the total cost of the intervention amounts to $261.70 per patient ($200 + 2 x $30.85).

If the same intervention is administered via an ACCHS, the additional health service components increase the cost of each consultation to $113.33 (see the Indigenous Health Service Delivery (IHSD) Template, Additional file 1) to bring the total cost of the intervention to $426.66, assuming that medication costs remain the same ($200 + 2 x $113.33).

Determining the magnitude of the weight:
Using these costs, the magnitude of the weight could be determined as the ratio of the two amounts ($261.70/$426.66) to give an equity weight of 0.61. In practice, the weight would be established using the average costs across multiple pharmacological interventions rather than a single case study.

Applying the weight:
The weight could be applied to the costs of all similar pharmacological interventions which are delivered to Indigenous populations from ACCHSs. For example, if the costs for a separate intervention were determined to be $100 per patient, then applying the weight ($100 x 0.61) would leave a weighted cost of $61 per patient which would then subsequently be used to determine the cost-effectiveness ratio. This weight would therefore adjust downwards the additional costs associated with the provision of targeted services for Indigenous populations, and thus allow equitable comparison with mainstream interventions within efficiency-based resource allocation formulae. For a hypothetical patient population of 100, for which the intervention produces 10 quality-adjusted-life-years (QALYs) of benefit, the resulting cost effectiveness ratio would change from the unweighted result of $1000 per QALY, to $610 per QALY. Note that intervention costs will need to be converted back to their unweighted form for use in determining funding allocations.
